# Phasic REM: Across Night Behavior and Transitions to Wake

**DOI:** 10.3390/brainsci15080840

**Published:** 2025-08-06

**Authors:** Giuseppe Barbato, Thomas A. Wehr

**Affiliations:** 1 Dipartimento di Psicologia, Università Degli Studi Della Campania “Luigi Vanvitelli”, 81100 Caserta, Italy; 2 NIMH Intramural Research Program, Bethesda, MD 20892, USA; wehr.tom@gmail.com

**Keywords:** REM sleep, REM activity REM phasic, REM density, REM tonic, wake

## Abstract

Background/Objectives: Rapid eye movements (REMs) during sleep were initially associated with dreaming, suggesting a relationship between REMs and dream content; however, this hypothesis was questioned by their differences with the REMs during wakefulness and the evidence that REMs are also present in blind individuals with no visual dreaming. Successive studies have focused on the phenomenology and physiological significance of REMs during sleep. REMs are categorized as expressions of the phasic REM component, which is characterized by bursts of eye movements, whereas the tonic REM component is characterized by quiescent periods without eye movements. Methods: The study is a retrospective analysis of 105 sleep records from 15 subjects. We analyzed the two components, tonic and phasic REM, across the sleep period, the REM activity in the first 5 min and in the last 5 min of each REM period were also assessed. Results: Phasic epochs were more represented than tonic epochs across the whole night period. REM activity in the first and last five minutes of an REM period presented different, although non-significant, patterns across the night. REM activity in the first 5 min showed a curvilinear profile, whereas REM activity in the last 5 min showed a linear increasing trend. A significant correlation was found between the REM activity in the first 5 min of the REM period and the total duration of the REM period. Conclusions: According to our results, the analysis of REM activity and the focus on segments of an REM period could provide more information both on the temporal evolution of REM activity within an REM period and on the possible role of REMs in REM sleep regulation and its significance in psychiatric and neurological disorders.

## 1. Introduction

After the breakthrough paper of Aserinski and Kleitman [1] that described periods of rapid conjugate eye movements occurring during sleep and associated with recall of dream content, the acronym REM (rapid eye movement) was first used by Dement and Kleitman [2], which also described the cyclic occurrence of REM sleep across the night.

Rapid eye movements (REMs) were initially associated with dreaming, suggesting a relationship between REMs and dream content [3] in that REMs were supposed to follow the dream content, “scanning” what was happening in the dream.

Recent data from Andrillon et al. [4] and Senzai and Scanziani [5] have revitalized the “scanning hypothesis” by showing a relationship between REMs and brain events associated with waking vision; however, the hypothesis that REMs are indicative of visual imagery of the occurring dream is questioned by several observations. Reports of visual dreaming are also evident during NREM sleep and during REM sleep in absence of REMs, thus rapid eye movements do not necessarily lie behind the dreaming process. Differences between the rapid eye movements during wakefulness and those occurring during the REM periods have been highlighted in several studies (see review of Arnulf [6] and Moskovitz and Berger [7]). Furthermore, REMs during REM sleep are also present in blind individuals who report no visual dreaming [8], spontaneous REMs have been found in a hydranencephalic infant where the cortex was presumably absent [9], and REM behaviors have also been reported in a microcephalic infant [10], suggesting that cerebral structures are not involved in REMs.

Successive studies have focused on the phenomenology and physiological significance of REMs, as well as the differences in REMs in pathological conditions [11,12,13,14].

REMs are categorized as expressions of the phasic REM component, characterized by bursts of eye movements, whereas the tonic REM component is characterized by quiescent periods without eye movements [15,16]. REM bursting characteristics show a different behavior across the night sleep, bursts in the late REM periods tend to be longer and have more REMs compared to bursts in the early REM periods [17].

It has been proposed that the occurrence of REMs is not a random process but could result from the action of a periodic generator [12,18]. Aserinsky [17] reported that eye movement peaked 5–10 min after the onset of the REM period and then decreased 10 min later. The data also suggested the existence of a cyclic pattern, with REM periods of 40 and 60 min showing, respectively, two and three peaks of REMs activity. Salzarulo [19] reported that during an REM phase, frequency of eye movements increases progressively, reaches a peak value at the middle of the phase and decreases at the end; the variations also depend on the length of the phase and its location within the night. Peaks tend to be reached more rapidly in the longest phase.

Differences in neural states between phasic and tonic periods with respect to environmental alertness, cortical activity [20,21], information processing have also been suggested (see review of Simor et al. [15]), highlighting the physiological significance of the two components. External information processing appears to be attenuated, and cortical activity detached from the surrounding environment during periods of phasic activity [22], whereas environmental alertness is partially maintained during tonic periods [23,24].

To monitor phasic REM, different measures of REMs have been adopted: “REM activity” defines the number of REMs during an REM period, frequency of REMs is defined as “REM density”, reflecting the relationship between numbers of REMs (REM activity) and REM duration.

Although widely used in early studies, description of REM activity has been later dismissed, to favor REM density as the main index of REM phasic intensity, furthermore no study, as far as we know, has explored the temporal distribution and characteristics of the tonic and phasic components of REM sleep.

To further define the significance of REMs, we analyzed the tonic and phasic REM components across the sleep period. The REM activity in the first 5 min and in the last 5 min of each REM period were also analyzed. Finally, we compared the REM activity of REM periods that terminated with transitions to wakefulness with those of REM periods with transitions to stable sleep (stage 2) or intermediate, light sleep (stage 1).

## 2. Materials and Methods

The study is a retrospective analysis of 7 consecutive days of baseline condition (16 h light, 8 h dark) of 15 healthy volunteers (1 female, 14 males), for a total of 105 sleep records. Subjects participated in a 4-week short photoperiod study (10 h light, 14 h dark), two of them, one male and one female were exposed to the short photoperiod for 15 weeks [25]. Volunteers were screened with interviews, physical examinations, and routine laboratory tests and procedures. None had medical or psychiatric illnesses, and none had taken any medication for at least 3 weeks before the studies. The protocol was approved by the NIMH Intramural Research Project, and written informed consents were obtained.

Polysomnographic sleep was monitored with a Grass 78 D polygraph, using conventional electrode montage: C3-A2 and C4-A1 EEG channels, left and right EOG, and submental EMG. Sleep stages were visually scored for each 30 s epoch according to the criteria of Rechtschaffen and Kales [26]. An REM period was defined according to Rechtschaffen and Kales [26] and the start and end of an REM period were defined according to Rechtschaffen and Kales rules (page 9–11 of Ref. [26]). After visual scoring, data were recorded in Excel files and further processed with Excel’s macros for the different sleep and REM variables. A minimal duration of 5 min was adopted to define an REM period [27,28].

REM activity for each 30 s epoch of REM sleep was expressed on a 0–8 scale according to the number of eye movements (EMs): 0 corresponded to no EMs, 1, 1–2 EMs; 2, 3–5 EMs; 3, 6–9 EMs; 4, 10–14 EMs; 5, 15–20 EMs; 6, 21–26 EMs; 7, 27–32 EMs; 8, 33 and over EMs [29].

Tonic epochs were defined as epochs with no EMs or single isolated EM (epochs 0 or 1 on the activity scale), phasic epochs were defined as epochs with EMs (2–8 on the activity scale).

Following the end of an REM period, defined by the occurrence of a single epoch of any other stage, three possible conditions were classified: wake—period with transition to a stage 0 epoch (wake); intermediate—period with transition to a stage 1 epoch; sleep—period with transition to a stage 2 epoch.

Further analyses were conducted on REM activity during the first and last 5 min of each REM period.

For each subject, REM variables were averaged over 7 nights for each consecutive REM cycle across the night. For all subjects averages of the first three cycles were computed from all 7 nights; averages of the fourth cycle were computed from 7 nights for 11 subjects, from 6 nights for 2 subjects, and from 5 nights for the last two subjects; averages of the fifth cycle were computed from 7 nights for 3 subjects, from 6 nights for 4 subjects, from 5 nights for 1 subjects, and from 2 nights for 3 subjects; the data of the subject with only 1 night fifth cycle was not included in the analysis and 3 subjects did not show a fifth cycle.

To compare the tonic and phasic epochs, the different segments of the REM period (first 5 min and last 5 min), and their temporal characteristics and interactions, the data were analyzed using three separate between subjects factorial ANOVAs with the factors: (a) REM type of epochs (tonic and phasic) and cycles; (b) type of REM segment (first five minutes and last five minutes) and cycles; and (c) type of REM segment (first five minutes and last five minutes) and type of transitions (wake, intermediate, sleep). Linear regression on subject’s aggregated data was used for the analysis of the relationship between segments of REM activity within an REM period and the total REM duration of an REM period. Statistical analyses were conducted using STATISTICA 10. StatSoft, Inc. (Tulsa, OK, USA).

## 3. Results

**Figure 1:**
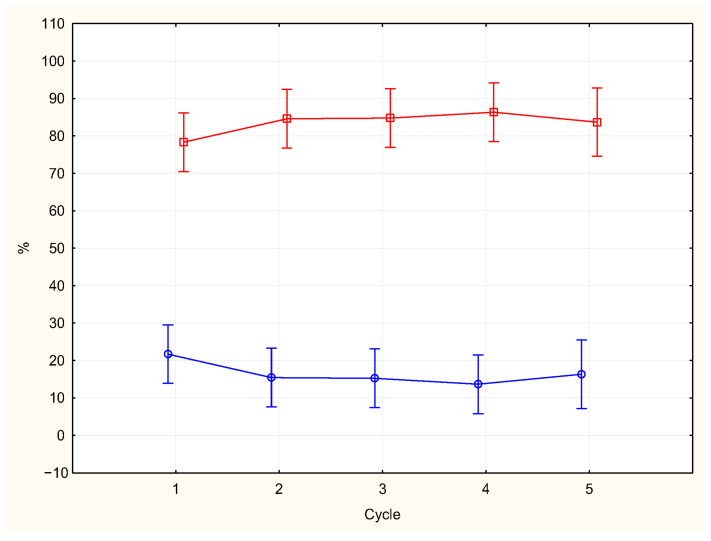
Percentages of epochs with tonic activity (blue line) and phasic activity (red line) across the night (vertical bars show SD).

REM time and REM density for each cycle are shown in Table 1. Phasic epochs were more represented than tonic epochs [83.9% (SD = 15.13) vs. 16.01% (SD = 15.13)] across the whole night period (F (1, 132) = 667.46, *p* = 0.0000), with no interaction effect of cycles and types of epochs (F (4, 132) = 1.20, *p* = 0.31). The percentage of phasic epochs showed a modest increasing trend towards the end of the night, the opposite trend was observed for the percentage of tonic epochs, with more tonic epochs in the first cycle (Figure 1); however, the respective percentages of phasic epochs and that of tonic epochs did not show significant differences across the night.

**Figure 2:**
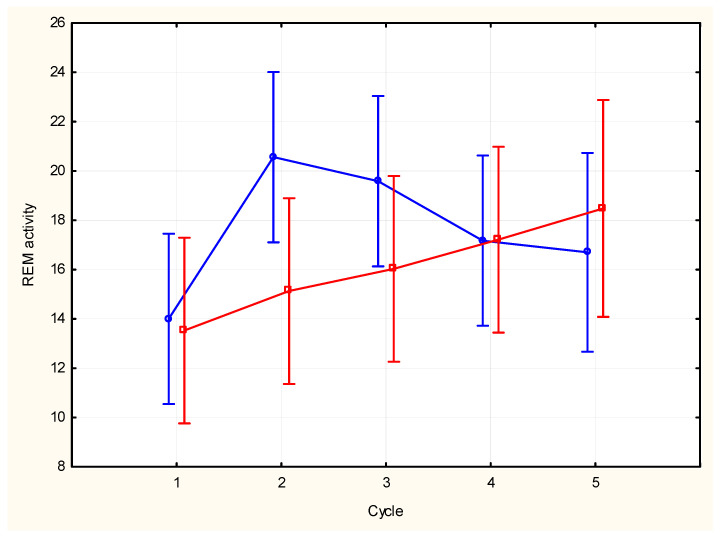
Comparison across the night of first (blue line) and last (red line) five minutes of REM activity in the REM periods (vertical bars show SD).

**Figure 3:**
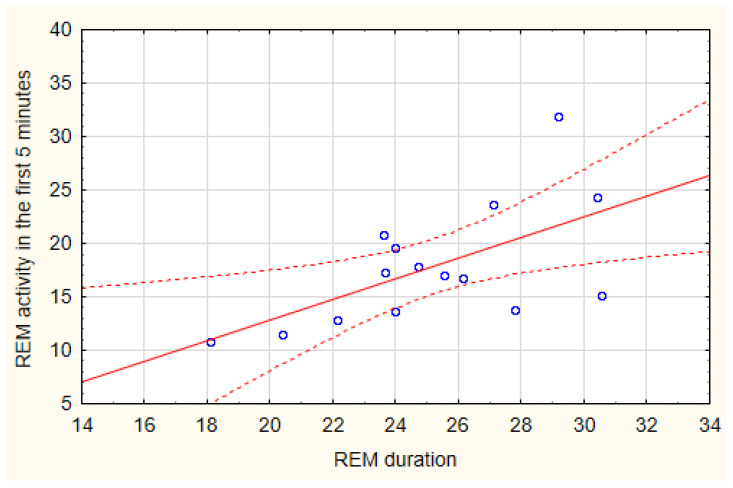
Scatterplot of the correlation between REM activity during the first five minutes of an REM period and the total duration of the REM period.

REM activity in the first and last five minutes of an REM period showed non-significant differences across the night (type of REM segment: F (1, 132) = 1.19, *p* = 0.27; cycle: F (4, 132) = 1.93 *p* = 0.11; cycle x type of REM segment: F (4, 132) = 1.17, *p* = 0.33) (Figure 2). A significant correlation was found between the activity of the first 5 min of the REM period and the total duration of the REM period (N = 15, R^2^ = 0.369, *p* = 0.016) (Figure 3), no significant correlation was found between the activity of the last five minutes of the REM period and the total duration of the REM period (N = 15, R^2^ = 0.043, *p* = 0.46). Analysis of correlation between activity in the first five minutes of an REM period and the total duration of the REM period at each time point showed positive correlations that was significant only in the first cycle (Cycle 1: R^2^ = 0.41, *p* = 0.01; Cycle 2: R^2^ = 0.21, NS; Cycle 3: R^2^ = 0.25, NS; Cycle 4: R^2^ = 0.04 NS; Cycle 5: R^2^ = 0.02, NS).

**Figure 4:**
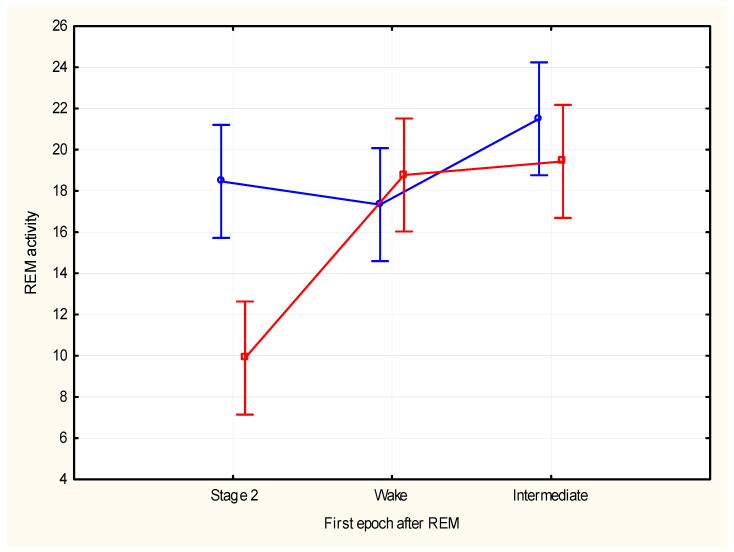
REM activity of first (blue line) and last (red line) five minutes of total REM activity preceding transitions to stage 2, wake, or stage 1. (vertical bars show SD).

The REM density was not significantly different for REM periods with transition to wake or to stage 1 or to stage 2 (F (2, 42) = 1.73, *p* = 0.19). Total REM activity was not significantly different for REM periods with transition to wake or to stage 1 or to stage 2 (F (2, 42) = 2.80, *p* = 0.072). Analysis of REM activity in the first and last five minutes segments showed significant differences according to the type of segment (F (1, 84) = 7.43, *p* = 0.008, η^2^ = 0.08) and different types of transitions (F (2, 84) = 10.60, *p* = 0.00008, η^2^ = 0.20), and also showed significant interaction between type of transitions and type of REM segment (first vs. last) (F (2, 84) = 6.79, *p* = 0.002, η^2^ = 0.14). REM activity during the first five minutes of the REM period was not significantly different for REM periods with transition to wake or to stage 1 or to stage 2 (F (2, 42) = 0.85, *p* = 0.43). REM activity during the last five minutes of the REM period was significantly different for REM periods with transition to wake or stage 1 compared to periods with transition to stage 2 (F (2, 42) = 13.59, *p* = 0.0001); post hoc analyses of activity during the last five minutes segments showed the following results according to the sleep transitions: wake vs. stage 2 (21.22 vs. 11.87, F (1, 28) = 26.40, *p* = 0.0001 Bonferroni corrected); wake vs. stage 1 (21.22 vs. 19.50, F (1, 28) = 0.60, *p* = 0.44); and stage 1 vs. stage 2 (19.50 vs. 11.87, F (1, 28) = 21.17, *p* = 0.0001 Bonferroni corrected) (Figure 4).

## 4. Discussion

In his “Harvey Lecture”, Giuseppe Moruzzi stated: “(…) Summing up, a distinction between underlying tonic changes and phasic outbursts is likely to be useful in any attempt to unveil—through more refined and more complete electrophysiological and behavioral analysis—the basic nature and the functional significance of sleep” [30].

The significance of phasic REM during REM sleep is not well understood, also it is not clear if the frequency of REMs (REM density) can be considered a measure of the intensity of REM sleep. After selective REM deprivation, increased pressure for REM in the recovery night is manifested by a decreased REM latency and increased REM percent, but not by an increased REM density, which instead is reduced [31].

Increased REM percent and REM density have been reported in depression and considered, together with shortened REM latency, as index of increased propensity (and/or pressure) for REM sleep in this clinical condition.

REM time and REM density do not show the same profile across the night. Aserinsky [32] found that in extended sleep, REM density increased with each successive REM episode; in contrast, REM episodes progressively increased across the first four episodes, then decreased in the fifth and sixth. Zimmermann et al. [33] studied the sleep of subjects in a free running condition and compared it with sleep in the entrained condition. REM density increased across successive REM episodes in both conditions, while REM sleep showed a phase advance in its rate of accumulation, with a longer duration of the earlier episodes in the free running condition compared with the entrained condition. Different mechanisms appear to regulate REM occurrence and its duration, and REM density. REM sleep time is controlled by short- and long-term homeostatic regulations [34,35,36], and by a definite circadian modulation which coincides with the body temperature minimum [37,38]. REM density increases as a function of time in consecutive sleep cycles, opposite to the decreasing NREM sleep pressure, and it has been associated with arousal during sleep [39,40,41]; it also presents a circadian modulation independent from the circadian modulation of REM sleep where the REM density peak occurs earlier in the circadian cycle [42].

A critical aspect in assessing significance and regulation of phasic REM is the lack of univocal and consistent methodologies used. REM density has been reported in several ways [43], with no consensus on a definite and reproducible way to measure it. Most studies measure REM density as the relationship between the total REM activity and the duration of the REM period. Other studies have instead considered REM density as the percentage of REM epochs with REMs (number of intervals that contained REMs).

Moreover, due to difficulties counting the large number of REMs that occur during a time unit, REM activity is mostly measured, using the Pittsburgh method, on a 9 points scale from 0 (no EMs) to 8 (more than 33 EMs) [44].

Few studies have adopted automatically measured scoring of REMs [45,46,47,48,49]. Automated analyses have also proposed different parameters, like EM directional characteristics [50], EM frequency, and EM rotation with speed and energy, showing a different behavior of the REMs across the night compared to traditional measures, with an inverted V pattern and higher EM rotation in the second cycle [45], and suggested fluctuation at a periodicity of about 2 min that may relate to rhythmic component of the REM generating mechanisms [46].

In our study, tonic and phasic epochs did not show a specific individual pattern across the night and epochs with bursts of eye movements were more frequent compared to the tonic epochs, but neither the tonic nor phasic epochs were significantly prevalent in a defined cycle.

Previous data on the behavior of REMs during the REM period have shown different results. According to Aserinsky [17] the total numbers of REMs are similar in both halves of an REM period, whereas for Petre-Quadens [51] the number of REMs tends to increase from the beginning to the midpart, then to decrease toward the end.

REM activity in the first and last five minutes of each REM period across the night presented different, although not statistically significant, patterns. A trend for a curvilinear profile was shown for REM activity in the first 5 min, whereas REM activity in the last five minutes showed a linear increasing trend. The REM activity in the first five minutes appeared to predict the length of the whole REM period and higher activity leading to a longer period, consistent with early observations of Salzarulo [19] who reported that peaks of EM density tend to be reached more rapidly in the longest phases, a data which suggests a possible relationship between the systems that control the phasic activity and those controlling the duration of the REM period. The positive relationship was more evident in the first cycles of the night, further suggesting that REM can be regulated by different mechanisms across the night period [34].

Considering that REM sleep and slow wave sleep can be competing variables within sleep [52,53], higher REM activity at the beginning of an REM period can express a higher REM propensity that would lead to a longer duration of the period.

The higher REM activity of the last five minutes was associated with transitions to epochs of stage 1 or wake, consistent with previous data of a relationship of increased REM density with transition to wake in extended sleep [28]. It has been suggested that REM density can be related to arousal during sleep [41] and increased phasic activity at the end of the REM period can possibly reflect a decrease in sleep pressure, leading the transition to a lighter sleep phase and/or to wake. Data on cortical excitability during phasic REM appears consistent with the suggested relationship with arousal. Usami et al. [20] reported that cortical excitability to exogenous input during phasic REM was directed toward wakefulness, which may produce incomplete short bursts of consciousness, leading to dreams.

## 5. Conclusions

The present study did not show specific REM cycle patterns of the tonic and phasic epochs, and phasic activity as reflected by bursts of REMs was always prevalent across the whole night period. Previously assessed only in a limited number of night’s samples [11,17,18,19], the analysis of temporal characteristics of phasic activity within an REM period has shown interesting differences between the early and late segments. The first five minutes of REM activity in the REM period appears to correlate with the total duration of the REM period and might have a role in REM regulation, whereas the last five minutes might be regulated by arousal mechanisms, suggesting that also within the REM period, different regulatory factors might control REM sleep.

Considering the retrospective and exploratory nature of the study, with no use of an experimental model to test the hypothesis, our results should be interpreted cautiously. Furthermore, for the REM activity categorization, we used a visual scale previously adopted in the REM literature [29,44]; although widely used, such scale does not provide the absolute number of REMs, nor information on direction and amplitude of REMs. Future studies should possibly implement the use of automated REMs detection and scoring [49,50], that could add information not accessible with simple visual scoring, and thus provide further insights to better understand the possible cognitive role of REMs [15] and their significance in dreaming processes and psychosis [54,55,56,57].

The results also confirmed a great inter-individual as well as intra-individual variability of the REM measures, which underscores the fact that the sleep cycle, even in controlled laboratory condition, is influenced by several factors that can contribute to a high variability of the different measures.

According to our results, the analysis of REM activity and the focus on segments of an REM period could provide information both on the temporal evolution of REM activity within an REM period and on the possible role and significance of REMs in REM sleep regulation.

## Figures and Tables

**Table 1:** Means REM time (minutes) and REM density.

Cycle (N)	REM Time (SD)	REM Density (SD)
1 (15)	16.41 (7.48)	1.71 (0.66)
2 (15)	27.35 (8.66)	2.15 (0.70)
3 (15)	30.98 (6.26)	2.27 (0.72)
4 (15)	25.63 (9.27)	2.16 (0.60)
5 (11)	26.78 (12.45)	2.11 (0.76)

## Data Availability

The original contributions presented in this study are included in the article. Further inquiries can be directed at the corresponding author.
